# Association and optimization of the Planetary Health Diet Index with hyperuricemia

**DOI:** 10.3389/fnut.2025.1657218

**Published:** 2025-09-15

**Authors:** Jia-xin Tan, Zhen Qin, Qing-zhong Li, Guo-tian Ruan, Yi-zhen Gong

**Affiliations:** ^1^Department of Clinical Research, Guangxi Medical University Cancer Hospital, Nanning, Guangxi, China; ^2^The First Affiliated Hospital of Guangxi Medical University, Nanning, Guangxi, China; ^3^Department of Neurosurgery, Guangxi Medical University Cancer Hospital, Nanning, Guangxi, China; ^4^Department of General Surgery, Beijing Friendship Hospital, Capital Medical University, Beijing, China

**Keywords:** Planetary Health Diet Index, hyperuricemia, NHANES, environmental protection, machine learning, restricted cubic splines

## Abstract

**Background:**

Hyperuricaemia (HUA) is a common metabolic disease that has become a global public health burden. The Planetary Health Diet Index for the United States (PHDI-US), a sustainable dietary pattern emphasizing a plant-based diet, has great potential for chronic disease prevention and control, but its relationship with HUA is unclear.

**Methods:**

This study was based on nationally representative NHANES data. Multivariate logistic regression and restricted cubic spline models were used to assess the association between raw PHDI-US and HUA. The key dietary component Dairy was identified by machine learning, the optimized PHDI-Dairy index was constructed, and the association between dairy intake and HUA risk was verified in an independent self-built cohort.

**Results:**

Primitive PHDI-US was significantly negatively correlated with the risk of HUA, but its protective effect was weaker than traditional dietary indices such as AHEI, AEI, and MEDI. Machine learning results show that Dairy is the most critical component in PHDI-US composition. The PHDI-Dairy index, constructed after optimizing the weight of dairy products, was more negatively correlated with the risk of HUA, and the protection efficiency was better than that of other dietary indices. External validation further confirmed that increased dairy intake was associated with a low risk of HUA.

**Conclusion:**

PHDI-US was negatively correlated with the risk of HUA. After optimizing the weighting of dairy products, PHDI-Dairy demonstrated a significantly enhanced protective effect, outperforming both the original PHDI and other dietary indices. This result highlights its potential as a nutritional intervention tool for chronic diseases, contributing to both public health and environmental sustainability.

## 1 Introduction

Hyperuricemia (HUA) refers to the pathological state in which the concentration of serum uric acid (SUA) is continuously higher than the normal threshold. This threshold is based on the saturated concentration of uric acid in physiological fluids. When it exceeds, the risk of urate crystal precipitation increases significantly, which can easily cause gout (1). The essence of HUA is the result of a purine metabolism disorder or renal excretion dysfunction (2). HUA is closely related to many chronic diseases, such as cardiovascular disease, renal dysfunction, and diabetes, and is an important risk factor for the occurrence and development of these diseases (3–5). With the development of the global economy and changes in people’s lifestyles, such as increased intake of high-purine foods in the diet, reduced exercise, and increased drinking, the incidence of HUA is increasing year by year. According to the data, the prevalence of hyperuricemia among adults in 31 provinces (autonomous regions and municipalities directly under the central government) in mainland China showed an upward trend; that is, the overall prevalence in 2018–2019 was 14.0%, which was 2.9 percentage points higher than 11.1% in 2015–2016 (6). The onset and progression of HUA are usually hidden. Many patients have no obvious symptoms in the early stage of the disease, and are often diagnosed when gout attacks or other complications occur. However, once complications occur, the difficulty of treatment will increase and the prognosis will be affected. Therefore, timely identification of HUA and early intervention are of great significance for preventing complications and reducing the burden of disease (7).

In recent decades, the global burden of diet-related non-communicable diseases (NCDs) has increased dramatically, and the current food production and consumption system also poses a major threat to the health of the planet. Based on this, the EAT-Leaf Blade Committee has proposed a dietary model that combines health and sustainability goals. In order to evaluate people’s compliance with this dietary pattern, it is necessary to establish corresponding indicators. The Planetary Health Diet Index for the United States (PHDI-US) came into being (8). PHDI-US emphasizes increasing the intake of fruits, vegetables, whole grains, nuts, and beans, while reducing the consumption of red meat, processed meat, and eggs. Studies have shown that following the dietary pattern advocated by PHDI-US is not only closely related to reducing the incidence of major diseases such as cancer (9), cardiovascular disease (10), and lung disease (11), but also can significantly reduce the negative impact on the environment. In contrast, other dietary indices such as the Alternative Healthy Eating Index (AHEI), the Healthy Eating Index (HEI), and the Mediterranean Diet Score (MEDI) have also been shown to be closely related to a variety of diseases (12). The difference is that most of these traditional dietary indexes only focus on the health benefits of diet and lack consideration of the environmental impact of dietary patterns, which makes it difficult to meet the current needs of coordinated development of health and environment. It is worth noting that dietary structure is closely related to the occurrence and development of HUA, and excessive intake of high-purine foods is one of the important factors leading to elevated serum uric acid (6). However, there is a paucity of studies that combine the PHDI-US with HUA to investigate the effects of healthy and sustainable dietary patterns on HUA. It is also unclear whether the PHDI-US is more advantageous than other dietary indices in reducing HUA. Therefore, the aim of this study was to investigate the association between PHDI and HUA, and to compare the protective effects of PHDI-US and other dietary indices (AHEI, HEI, MEDI) on HUA, and to find important components of PHDI-US to optimize PHDI-US, so as to provide a new perspective and theoretical support for improving HUA prevention and control strategies and promoting the realization of global health and environmental sustainable development goals.

## 2 Methods

### 2.1 Study population

**Figure 1:**
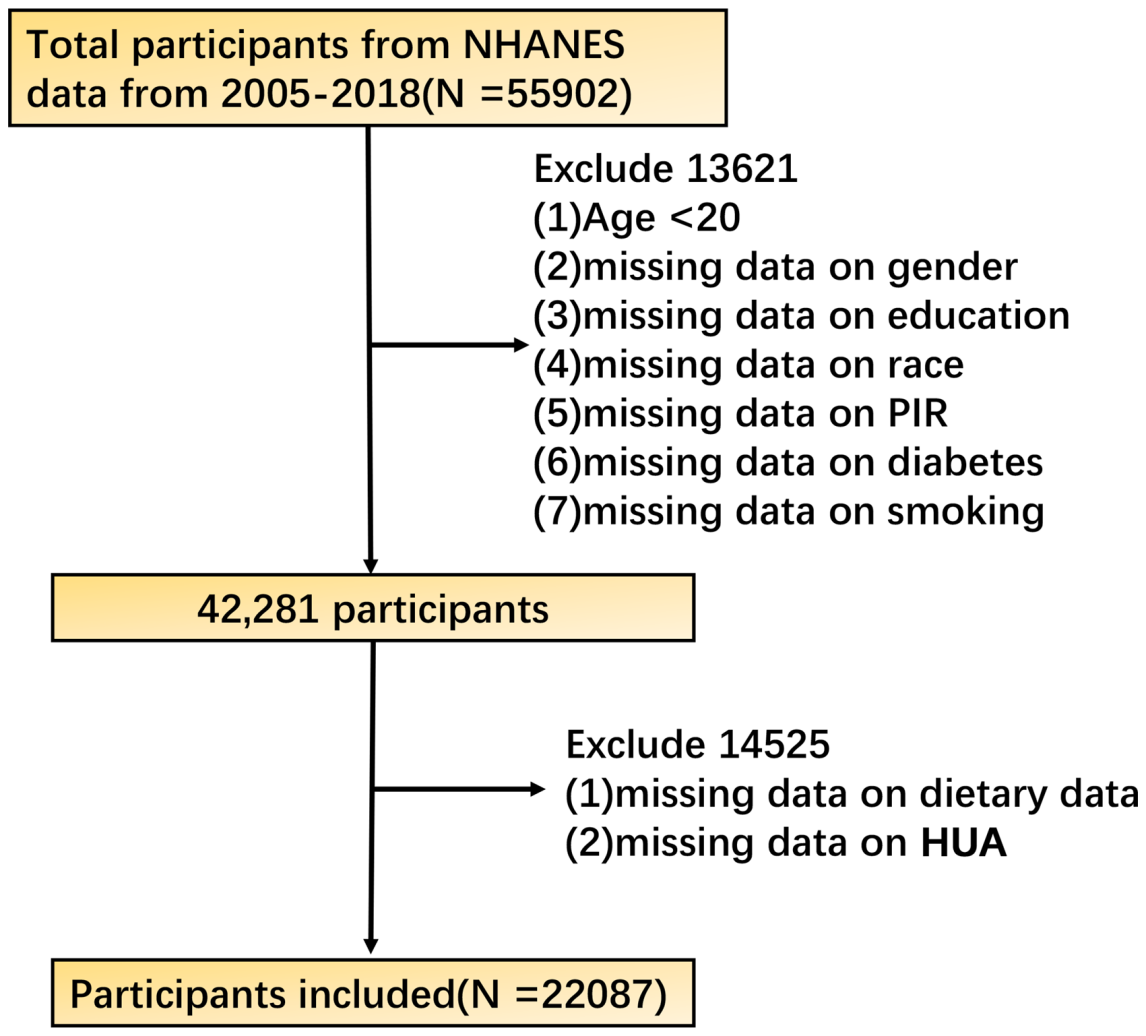
Flow chart of participant selection from the NHANES 2005–2018 cycles.

The data of this study are from the National Health and Nutrition Examination Survey (NHANES) public database. The NHANES aims to comprehensively assess the health and nutritional status of the U.S. population with a complex, stratified, multistage probability cluster sampling design. The study utilized data from the National Health and Nutrition Examination Survey (NHANES) with a sample size of 22,087 participants. Given that NHANES is a large, publicly available dataset, no explicit sample size estimation was required. The study selected the NHANES cycle data from 2005 to 2018 as the basis for analysis. The data during this period covered the latest diet, biochemical tests, and health status information. In terms of research object screening, adult participants aged ≥ 20 years were first included to ensure that the study population had relatively mature and stable characteristics in physiology and eating habits. Other exclusion criteria were: (1) missing data on gender, education, race, PIR, diabetes, and smoking; (2) Missing diet and HUA data. Finally, 22,087 participants were included in this study. The NHANES database has been approved by the Research Ethics Review Board of the National Center for Health Statistics of the United States, and all participants have signed informed consent to ensure the compliance and ethical rationality of research data acquisition. The research strictly follows the ethical principles established by the ‘Helsinki Declaration ‘from beginning to end. The presentation and publication of the research results are only displayed in the form of a group data summary, and do not involve the specific information of any individual participant. Relevant information can be obtained from the NCHS website (Figure 1).^1^

### 2.2 Assessment of Planetary Health Diet Index

Professionally trained investigators used the USDA Automated Multiple Pass Method to collect 24-h dietary review data (13). Participants were required to recall all the food and drinks consumed the previous day, and the measurement guidelines were used to assist in estimating food portions during the survey. 3–10 days after the first face-to-face interview, the investigators conducted a second unannounced dietary interview by telephone to obtain two-day dietary information and ensure the reliability and representativeness of the data. To incorporate dietary recall data into the Food Pattern Equivalent Database (FPED) (14). FPED assigns food to 37 U. S. Department of Agriculture food pattern components based on the food composition table. For a single component of food, direct distribution; for multi-component food, the standard recipe file is used to decompose it into the gram weight of each component to ensure the accurate classification of the data. The total energy intake (TEI) was derived from the two-day average total intake reported in the diet questionnaire and incorporated into all models to control confounding factors and reduce irrelevant variations in dietary variables, thereby providing a more accurate and reliable database for PHDI calculations (15). Finally, based on these processed data, combined with the PHDI scoring criteria, the participants ‘planetary health diet index scores were calculated. In this study, we used the R language tool DietaryIndex to calculate dietary indices such as PHDI-US, MEDI, AHEI, and HEI (16, 17). The source code, validation files, and tutorials are all open from GitHub,^2^ providing a standardized solution for dietary index calculation (18). PHDI-US consists of 16 components, with a total score of 0 to 150 points. It has six dimensions, including vegetables, fruits, whole grains, beans, nuts, healthy fats, and other plant foods intake, as well as the right amount of meat, fish, eggs, refined grains, and other foods (8). PHDI-US can quantify compliance with planetary healthy eating. The higher the score, the more in line with the dietary recommendations of a planetary healthy diet.

### 2.3 Diagnosis of hyperuricemia

Uric acid concentration was determined by the Beckman Coulter LX20 (Brea, CA) colorimetry (19). Hyperuricemia was defined as male ≥ 420 mmol/L (7 mg/dL), female ≥ 360 mmol/L (6 mg/dL), or use of uric acid-lowering drugs (20).

### 2.4 Self-built cohort

This study included a cohort of 2,819 individuals from southern China with complete data on hyperuricemia (HUA) and dairy intake. This cohort was a retrospective study conducted in Guangxi, where participants were selected based on the following criteria: adults aged over 20 years, with documented uric acid laboratory tests, physical examinations, and no clear history of renal disease, nephritis, or related endocrine disorders. The diagnostic criteria for HUA were consistent with those described above, and dairy intake was assessed based on the component-specific scoring standards of the Planetary Health Diet Index (PHDI) developed by the EAT–Lancet Commission. This was a retrospective study that did not involve any personally identifiable information or biological specimens. Ethical approval was obtained from the Ethics Committee of Liuzhou People’s Hospital (Approval No. KY2025-009-01). Research involving human participants was reviewed and approved by the same committee. All participants provided written informed consent prior to inclusion in the study.

### 2.5 Covariate

The covariates were determined by a comprehensive review of literature and clinical expertise. These variables include socio-demographic characteristics such as age, gender, race, education level, household poverty-to-income ratio (PIR), drinking and smoking history. In addition, hypertension and diabetes outcomes were also considered. Demographic characteristics were collected through standardized questionnaires and face-to-face interviews. With 1.3 times the poverty line as the boundary, PIR < 1.3 is defined as the poverty level, and PIR ≥ 1.3 is the non-poverty level. Hypertension was defined as a patient’s self-reported history of hypertension, use of antihypertensive drugs, systolic blood pressure (SBP) ≥ 140 mmHg, or diastolic blood pressure (DBP) ≥ 90 mmHg. Diabetes was defined as a history of diabetes, HbA1c level ≥ 6.5% or fasting blood glucose level ≥ 126 mg/dL. The classification of the remaining covariates can be seen in Table 1.

**Table 1:** Baseline characteristics of participants.

Characteristic	Non-HUA	With HUA	P value
PHDI-US	64.58 (±14.88)	63.87 (±14.68)	0.05
Gender			<0.001
Female	55,869,863 (46.85%)	14,369,471 (58.59%)	
Male	63,388,078 (53.15%)	10,156,729 (41.41%)	
Age			<0.001
20–45	59,754,823 (50.11%)	10,311,666 (42.04%)	
45–60	34,348,266 (28.80%)	6,549,745 (26.71%)	
60–75	19,274,327 (16.16%)	5,388,588 (21.97%)	
>75	5,880,525 (4.93%)	2,276,201 (9.28%)	
BMI			<0.001
<18.5	2,053,994 (1.72%)	38,979 (0.16%)	
18.5–25	38,911,117 (32.63%)	3,018,773 (12.31%)	
25–30	40,327,528 (33.82%)	7,395,285 (30.15%)	
>30	37,965,302 (31.83%)	14,073,163 (57.38%)	
Race			<0.001
Mexican American	10,005,584 (8.39%)	1,486,759 (6.06%)	
Other Hispanic	6,198,528 (5.20%)	956,172 (3.90%)	
Non-Hispanic White	83,689,523 (70.18%)	17,844,956 (72.76%)	
Non-Hispanic Black	11,188,947 (9.38%)	2,615,450 (10.66%)	
Other race—including multi-racial	8,175,359 (6.86%)	1,622,863 (6.62%)	
PIR			0.65
Below poverty threshold	36,726,064 (30.80%)	7,664,655 (31.25%)	
Above poverty threshold	82,531,878 (69.20%)	16,861,545 (68.75%)	
Education			0.01
Below high school	15,180,336 (12.73%)	3,043,215 (12.41%)	
High school	25,755,442 (21.60%)	5,977,814 (24.37%)	
Above high school	78,322,163 (65.67%)	15,505,171 (63.22%)	
Smoking			<0.001
No	50,491,804 (42.34%)	11,285,443 (46.01%)	
Yes	68,766,138 (57.66%)	13,240,757 (53.99%)	
Drinking			0.03
No	116,266,271 (97.49%)	23,716,987 (96.70%)	
Yes	2,991,670 (2.51%)	809,213 (3.30%)	
Hypertension			<0.001
No	118,489,056 (99.36%)	24,062,074 (98.11%)	
Yes	768,885 (0.64%)	464,126 (1.89%)	
Diabetes			<0.001
No	8,646,073 (7.25%)	2,780,238 (11.34%)	
Yes	110,611,868 (92.75%)	21,745,962 (88.66%)	

### 2.6 Machine learning

We applied a random forest regression (RFR) model to evaluate the relative importance of 16 dietary components in relation to hyperuricemia. The dataset was randomly split into a training set (70%) and a testing set (30%) to evaluate model performance. The model parameters were tuned to reduce overfitting risk: the number of trees was set to 100, the maximum tree depth was restricted to 3, the minimum samples required for splitting an internal node was 2, and the minimum samples required at a leaf node was 1. The loss function was set to squared error. The maximum number of features considered at each split was optimized, and the threshold for splitting was set at 0.0001. Model performance and generalizability were assessed using the testing set and out-of-bag (OOB) error estimates. The comparable performance between training and testing sets indicated that overfitting was not a major concern. Feature importance was subsequently derived from the mean decrease in impurity across all trees.

### 2.7 Statistical method

In order to ensure the national representativeness of the survey data, this study adopted the weight system recommended by the National Center for Health Statistics (NCHS). Continuous variables were described by mean ± standard deviation (normal distribution) or median (non-normal distribution), and t test was used to compare the differences between groups. The categorical variables were described by composition ratio, and the chi-square test was used for comparison between groups. Using the quartile grouping method, the subjects were divided into four groups according to the PHDI level, and the lowest group (Quartile 1) was used as a reference to construct three logistic regression models that gradually adjusted confounding factors. Model 1 did not adjust for confounding factors, Model 2 adjusted for gender and age, and Model 3 adjusted for age, gender, race, PIR, education, BMI, smoking, and hypertension. The odds ratio (OR) and 95% confidence interval (95% CI) were calculated to show the association strength between different dietary quality index groups and HUA, and the trend test (P for trend) was performed to determine whether the association changed regularly with the grouping.

At the same time, the restricted cubic splines (RCS) method was used to fit the dose–response relationship model, and the RCS Prediction Plot was drawn to visually present the nonlinear correlation between the continuous variables of dietary quality index and HUA risk. In order to identify the dietary factors most related to the reduction of HUA risk, machine learning methods such as random forest and feature importance analysis were used to evaluate 16 food components of PHDI-US. Based on the key features of model screening, the original PHDI-US is recalibrated. Then, through logistic regression and linear regression, the recalibrated PHDI-US, original PHDI-US, and the other three dietary indices (AHEI, HEI, and MEDI) were compared to analyze the difference in the efficacy of each index in reducing the risk of HUA and its linear correlation with HUA. In order to verify the dose–response relationship between dairy intake and hyperuricemia, this study used an independent cohort of the southern Chinese population for analysis, and used restricted cubic spline (RCS), logistic regression, and quantile regression to evaluate. It is worth noting that although the study confirmed that there was a significant correlation between PHDI-US and HUA, the results only showed correlation, and no causal relationship was established. A cohort of the southern Chinese population was used to verify the dose–response relationship between dairy intake and hyperuricemia. The statistical methods used were restricted cubic spline, logistic regression, and quantile regression. All statistical analyses were performed using the R language. All hypothesis tests were set to a bilateral significance level of 0.05; that is, when the *p*-value was less than the threshold, the results were considered statistically significant.

## 3 Results

### 3.1 Baseline characteristics of the study population

To explore the differences in characteristics between non-HUA and HUA populations and their potential association with PHDI-US, this study analyzed baseline characteristics by grouping patients with or without HUA. The baseline table was generated using NHANES survey weights with stratification and clustering to ensure nationally representative estimates. The results showed that (Table 1), the mean PHDI-US of the non-HUA group was 64.58 ± 14.88, which was higher than that of the HUA group (63.87 ± 14.68), but the difference between the groups did not reach a statistically significant level (*p =* 0.05). In the non-HUA group and the HUA group, the proportion of women was 46.85% vs. 58.59%, and the proportion of men was 53.15% vs. 41.41% (*p <* 0.001). The proportion of 20–45, 45–60, 60–75, and > 75 years old varied between the two groups, with statistically significant differences (*p <* 0.001). Specifically, the younger age group (20–45 years) was more prevalent in the no HUA group (50.11%), and the older age group (>75 years) was more prominent in the HUA group (9.28%). In terms of BMI categories, underweight (<18.5) and normal weight (18.5–25) were more common in the non-HUA group (1.72, 32.63%), whereas overweight (25–30) and obesity (>30) were more common in the HUA group (30.15, 57.38%), with statistically significant differences (*p* < 0.001). The HUA group had a higher proportion of Non-Hispanic White (*p* < 0.001). The proportion of current smokers in the HUA group was lower than that in the non-HUA group, and the proportion of drinkers was higher than that in the non-HUA group (*p <* 0.05). The non-HUA group had a higher proportion of people with lower educational levels (*p <* 0.05). In addition, the HUA group had a higher proportion of hypertension and a lower proportion of diabetes (*p <* 0.001). There was no statistically significant difference in economic level between the two groups (*p >* 0.05).

### 3.2 Association of PHDI-US/AHEI/HEI/MEDI with HUA

**Figure 2:**
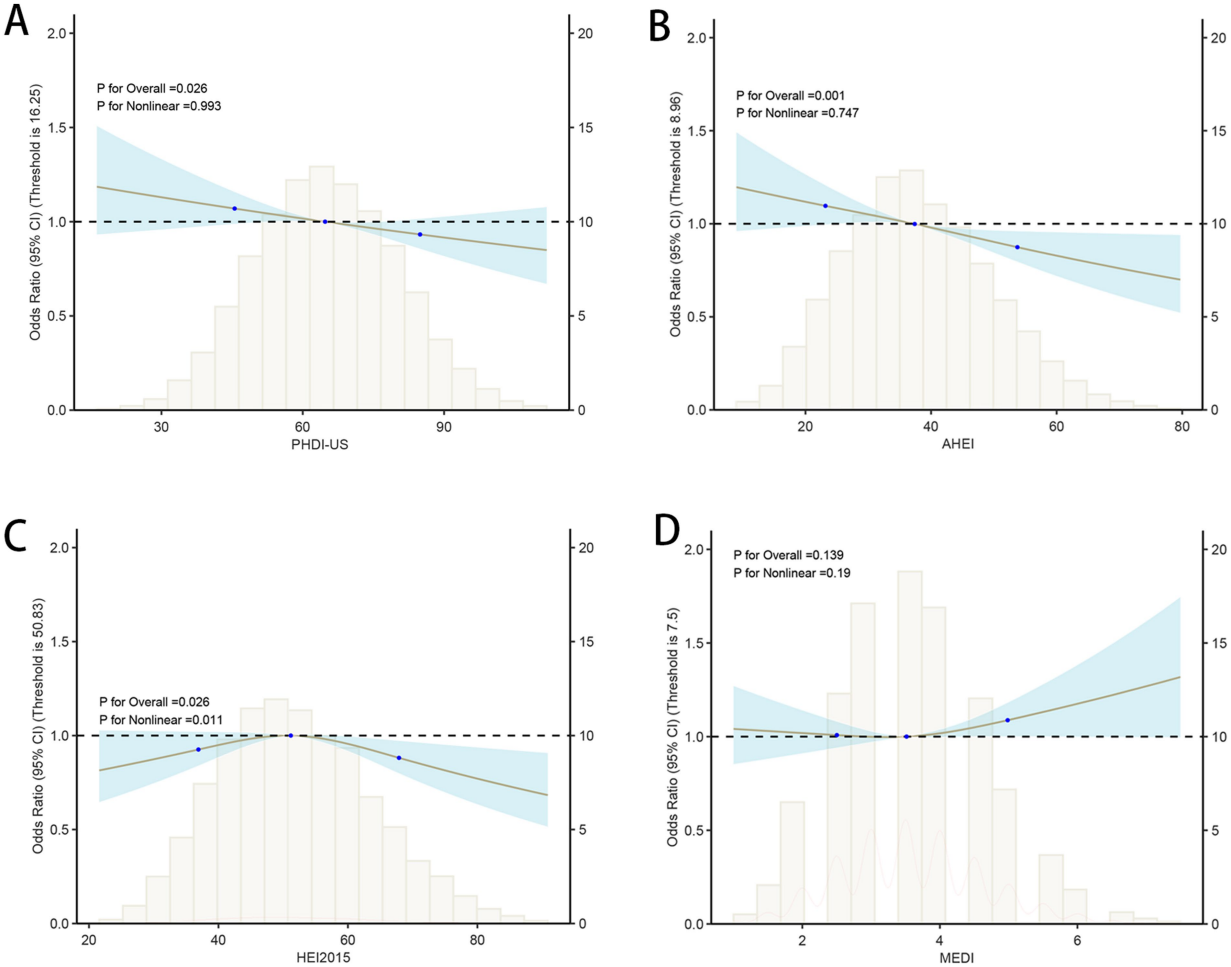
Associations between dietary quality indices and hyperuricemia (HUA) based on restricted cubic spline models. **(A)** PHDI-US; **(B)** AHEI; **(C)** HEI-2015; **(D)** MEDI.

In this study, model construction and RCS analysis were used to explore the association and dose–response relationship between dietary quality index (PHDI-US, AHEI, HEI, and MEDI) and HUA. The results showed that (Table 2), with the increase of PHDI quartiles, OR in Model 1–3 increased first and then decreased, and the trend test *p* < 0.01. In Model 3, the OR value of PHDI-US Q4 was 0.82 (95% CI, 0.73–0.93), which was lower than that of HEI Q4 (0.86, 95% CI, 0.76–0.97), but higher than that of AHEI Q4 (0.77, 95% CI, 0.67–0.87), indicating that the correlation between PHDI-US Q4 and HUA risk reduction was stronger than that of HEI but weaker than that of AHEI. The association between MEDI Q4 and HUA was statistically significant only in Model 2 (0.87, 95% CI, 0.77–0.99), showing a protective effect. In Model 1 and Model 3, the association was not significant (*p >* 0.05). RCS images further visualize the nonlinear dose–response relationship between dietary index and HUA risk. The results (Figure 2) showed that a significant decrease in the risk of HUA was observed with the increase of PHDI-US, AHEI, and HEI scores (*p* < 0.05). Compared with PHDI-US, elevated AHEI and HEI scores showed a stronger risk-reducing effect on HUA. In contrast, no statistically significant association between MEDI score and HUA risk was observed (*p* > 0.05).

**Table 2:** Association between dietary quality indices and HUA by quartiles.

Variable name	Model 1	*p*-value	Model 2	*p*-value	Model 3	*p*-value
	OR (95%CI)		OR (95%CI)		OR (95%CI)	
PHDI-US						
Quartile 1	Reference	-	Reference	-	Reference	-
Quartile 2	0.90 (0.81–1.01)	0.07	0.87 (0.78–0.95)	<0.01	0.86 (0.77–0.97)	0.02
Quartile 3	0.95 (0.85–1.07)	0.43	0.91 (0.82–1.03)	0.13	0.92 (0.82–1.03)	0.14
Quartile 4	0.83 (0.74–0.93)	<0.01	0.81 (0.73–0.91)	<0.01	0.82 (0.73–0.93)	<0.01
P for trend		<0.01		<0.01		<0.01
AHEI						
Quartile 1	Reference	-	Reference	-	Reference	-
Quartile 2	0.99 (0.89–1.11)	0.95	0.91 (0.81–1.02)	0.11	0.95 (0.85–1.07)	0.43
Quartile 3	0.93 (0.84–1.04)	<0.01	0.81 (0.73–0.91)	<0.01	0.89 (0.79–1.07)	0.06
Quartile 4	0.78 (0.70–0.87)	<0.01	0.62 (0.55–0.70)	<0.01	0.77 (0.67–0.87)	<0.01
P for trend		<0.01		<0.01		<0.01
HEI						
Quartile 1	Reference	-	Reference	-	Reference	-
Quartile 2	1.01 (0.91–1.13)	0.77	0.95 (0.85–1.06)	0.39	0.99 (0.88–1.11)	0.92
Quartile 3	0.97 (0.87–1.09)	0.65	0.88 (0.78–0.98)	0.02	0.99 (0.88–1.12)	0.95
Quartile 4	0.85 (0.76–0.95)	<0.01	0.70 (0.62–0.79)	<0.01	0.86 (0.76–0.97)	0.02
P for trend		<0.01		<0.01		<0.01
MEDI						
Quartile 1	Reference	-	Reference	-	Reference	-
Quartile 2	0.94 (0.85–1.06)	0.32	0.91 (0.81–1.02)	0.11	0.9 3 (0.82–1.04)	0.19
Quartile 3	0.90 (0.82–0.99)	0.04	0.86 (0.78–0.95)	<0.01	0.96 (0.86–1.07)	0.49
Quartile 4	0.96 (0.85–1.08)	0.52	0.87 (0.77–0.99)	0.04	1.06 (0.93–1.21)	0.41
P for trend		0.14		<0.01		0.74

Model 1, No covariates were adjusted; Model 2, Adjusted for gender and age; Model 3, Adjusted for age, gender, race, PIR, education, BMI, smoking, and hypertension. PHDI, Planetary Health Diet Index; AHEI, Alternative Healthy Eating Index; DII, Dietary Inflammatory Index; HEI, Healthy Eating Index; HEI, Healthy Eating Index. MEDI, Mediterranean Diet Index; OR, Odds Ratio; CI, Confidence Interval.

### 3.3 Machine learning identifies the relative importance of specific dietary components in PHDI-US

**Figure 3:**
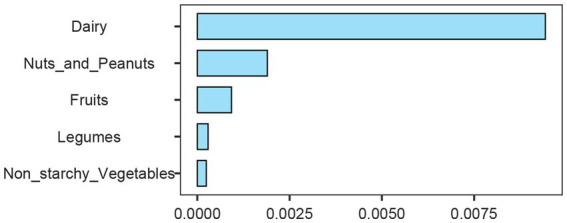
Importance ranking of dietary components based on random forest analysis. Dairy, nuts and peanuts, fruits, legumes, and non-starchy vegetables are shown according to their relative contribution.

**Figure 4:**
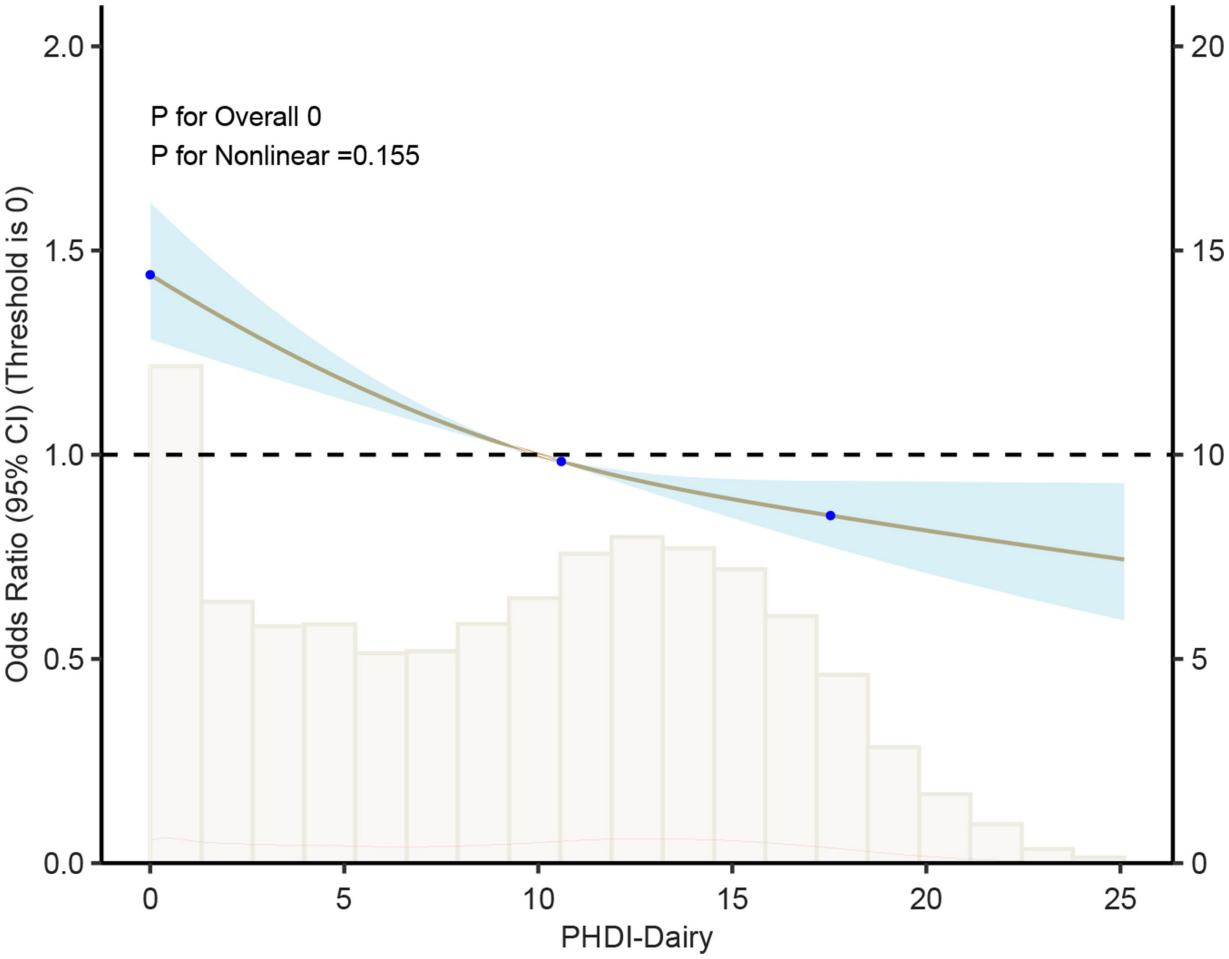
Association between PHDI-Dairy and hyperuricemia (HUA) based on restricted cubic spline models. The curve depicts odds ratios (95% CI) across PHDI-Dairy levels, with histograms showing the distribution of participants.

**Figure 5:**
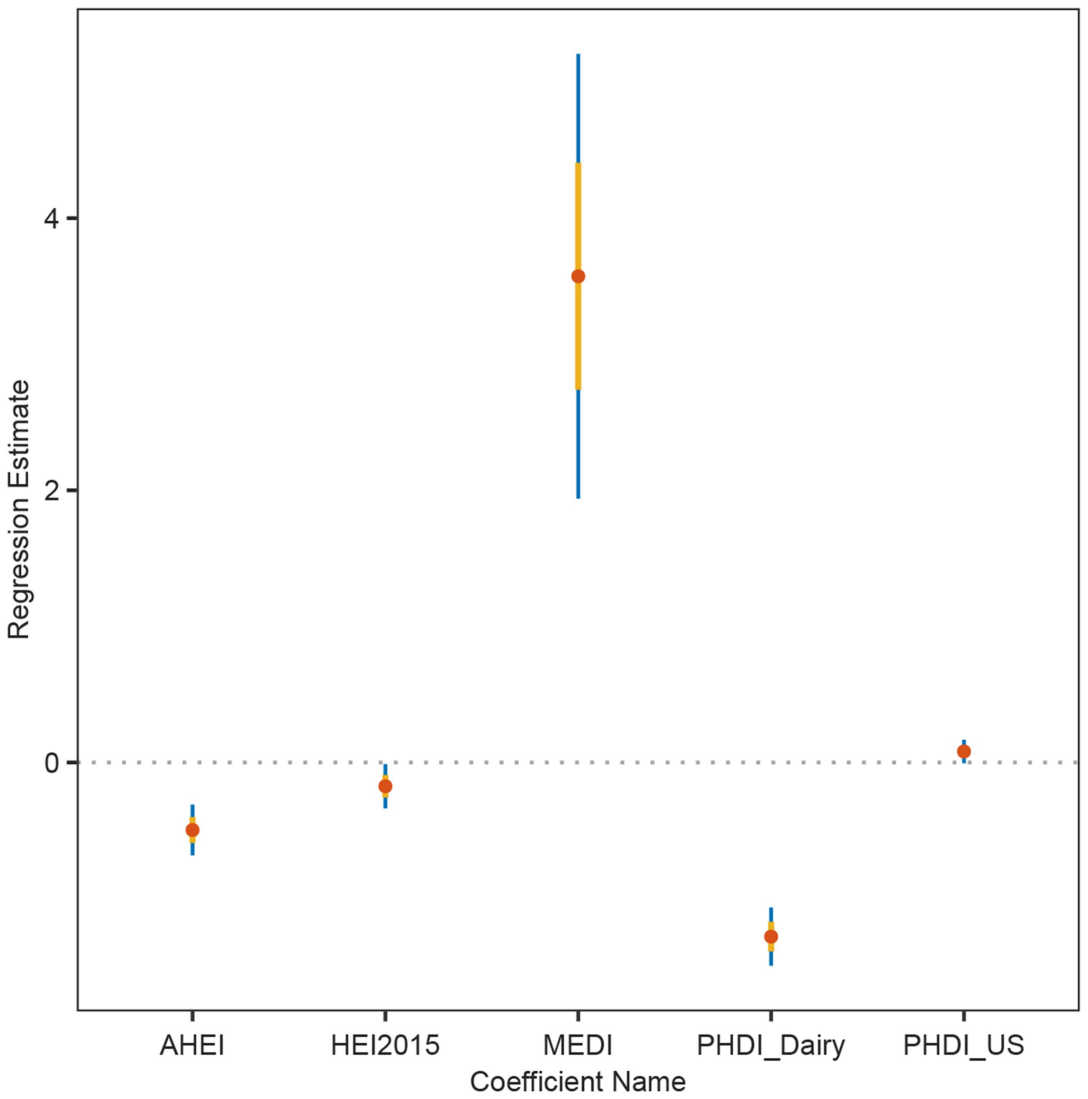
Linear regression coefficients of dietary indices in relation to hyperuricemia (HUA). Estimates and 95% confidence intervals were derived from multivariable linear regression models for AHEI, HEI-2015, MEDI, PHDI-Dairy, and PHDI-US.

In order to solve the problem that the correlation strength of PHDI-US in the HUA association study is lower than that of other dietary indices, this study introduces a random forest algorithm to mine the feature importance of 16 dietary components contained in PHDI-US. The results of feature importance analysis (Figure 3) showed that “Dairy” was the most influential dietary component associated with HUA risk. Based on this finding, this study constructed a modified index, PHDI-Dairy, by weighted adjustment and recalibration of dairy ingredients. Multivariate logistic regression was used to evaluate the association between PHDI-Dairy and HUA. The results (Table 3) showed that in the three models, with the increase of quartiles, the OR values associated with PHDI-Dairy and HUA gradually decreased (*p <* 0.05), indicating that adherence to PHDI-Dairy was associated with a reduced risk of HUA. Among them, after adjusting for all confounding factors (Model 3), the OR value of PHDI-Dairy fourth quartile (Q4) associated with HUA was 0.73 (95% CI, 0.65–0.82, *p* < 0.01), indicating that PHDI-Dairy has a strong negative correlation with HUA and is superior to other dietary indices. It is worth noting that at the fourth quantile level, the adjusted PHDI-Dairy increased the risk reduction benefit by 9% compared with the original PHDI (OR: 0.73 vs. 0.82). In order to further compare the correlation strength between the optimized PHDI-Dairy and other dietary indexes on HUA, this study used a multivariate linear regression model to evaluate the correlation between each dietary index and HUA. The results showed that (Table 4), PHDI-Dairy was negatively correlated with HUA risk, and the correlation was the strongest compared with other dietary indices (Estimate = −1.28, SE = 0.11, t = −11.71, 95% CI: −1.49~−1.07, *p <* 0.001). For every 1 unit increase in PHDI-Dairy, the risk of HUA decreased by an average of 1.28 points. In addition, AHEI and HEI were negatively correlated with HUA (*p <* 0.05). In contrast, PHDI-US was not significantly associated with HUA (*p >* 0.05), and MEDI was positively correlated with HUA (*p <* 0.05). The RCS results further revealed (Figure 4) that PHDI-Dairy was significantly negatively correlated with HUA risk (*P* for overall < 0.001) compared with PHDI-US before optimization. Linear regression coefficients showed that among all dietary indicators, the absolute value of the negative coefficient of PHDI-Dairy was the largest, indicating that it had the strongest correlation with HUA. The absolute value of the negative coefficient of HEI is small, and the absolute value of the negative coefficient of AHEI is greater than that of HEI, but weaker than the adjusted PHDI-Dairy (Figure 5). The above differences were statistically significant.

**Table 3:** Association of PHDI-Diary with HUA.

Variable name	Model 1	*p*-value	Model 2	*p*-value	Model 3	*p*-value
	OR (95%CI)		OR (95%CI)		OR (95%CI)	
Low PHDI-Dairy	Reference	-	Reference	-	Reference	-
High PHDI-Dairy	0.97 (0.96–0.98)	<0.01	0.97 (0.96–0.98)	<0.01	0.95 (0.94–0.97)	<0.01
Interquartile						
Quartile 1	Reference	-	Reference	-	Reference	-
Quartile 2	0.90 (0.81–1.01)	0.06	0.91 (0.82–1.02)	0.12	0.90 (0.81–1.02)	0.09
Quartile 3	0.71 (0.63–0.79)	<0.01	0.70 (0.63–0.79)	<0.01	0.74 (0.66–0.83)	<0.01
Quartile 4	0.69 (0.62–0.77)	<0.01	0.69 (0.61–0.78)	<0.01	0.73 (0.65–0.82)	<0.01
P for trend		<0.01		<0.01		<0.01

Model 1, No covariates were adjusted; Model 2, Adjusted for gender and age; Model 3, Adjusted for age, gender, race, PIR, education, BMI, smoking, and hypertension. HUA, hyperuricemia; OR, Odds Ratio; CI, Confidence Interval.

**Table 4:** Multiple Linear Regression Analysis of the Association Between Dietary Indices and Uric Acid.

Variable name	Estimate	Std_Error	t_value	*p* value	CI_2.5	CI_97.5
(Intercept)	344.30	3.91	88.09	0	336.64	351.96
PHDI-US	0.08	0.04	1.83	0.07	−0.01	0.17
PHDI-Dairy	−1.28	0.11	−11.71	0.00	−1.49	−1.07
AHEI	−0.50	0.10	−5.22	0.00	−0.68	−0.31
HEI-2015	−0.18	0.08	−2.11	0.03	−0.34	−0.01
MEDI	3.57	0.83	4.28	0.00	1.94	5.21

PHDI, Planetary Health Diet Index; AHEI, Alternative Healthy Eating Index; HEI, Healthy Eating Index; HEI, Healthy Eating Index; MEDI, Mediterranean Diet Index; CI, Confidence Interval.

### 3.4 Sensitivity analysis

Compared with PHDI-US, PHDI-Dairy optimizes the prediction performance to some extent. Specifically, the AUC of PHDI-US was 0.51 (95% CI: 0.50–0.52), which was close to the random level. The sensitivity of PHDI was high but the confidence interval was wide (0.72, 95% CI: 0.28–0.83). The specificity of PHDI-US was low (0.30, 95% CI: 0.21–0.76). The positive predictive value (PPV) was weak (0.18, 95% CI: 0.17–0.19), and the negative predictive value (NPV) was good (0.84, 95% CI: 0.83–0.86). The AUC of the modified PHDI-Dairy was slightly increased to 0.54 (95% CI: 0.53–0.55). The sensitivity decreased to 0.56 (95% CI: 0.53–0.58) but the confidence interval was significantly narrowed. The specificity was improved to 0.51 (95% CI: 0.50–0.52), and a good negative exclusion ability was maintained (NPV was 0.85, 95% CI: 0.84–0.86). More details can be seen in Table 5.

**Table 5:** Predictive Performance of PHDI and PHDI-Dairy for HUA.

Models	AUC (95% CI)	Sensitivity (95% CI)	Specificity (95% CI)	PPV (95% CI)	NPV (95% CI)
PHDI-US	0.51 (0.50–0.52)	0.72 (0.28–0.83)	0.30 (0.21–0.76)	0.18 (0.17–0.19)	0.84 (0.83–0.86)
PHDI-Dairy	0.54 (0.53–0.55)	0.56 (0.53–0.58)	0.51 (0.50–0.52)	0.20 (0.19–0.21)	0.85 (0.84–0.86)

AUC, area under the receiver operating characteristic curve; CI, confidence interval; PPV, positive predictive value; NPV, negative predictive value.

### 3.5 Independent cohort

**Figure 6:**
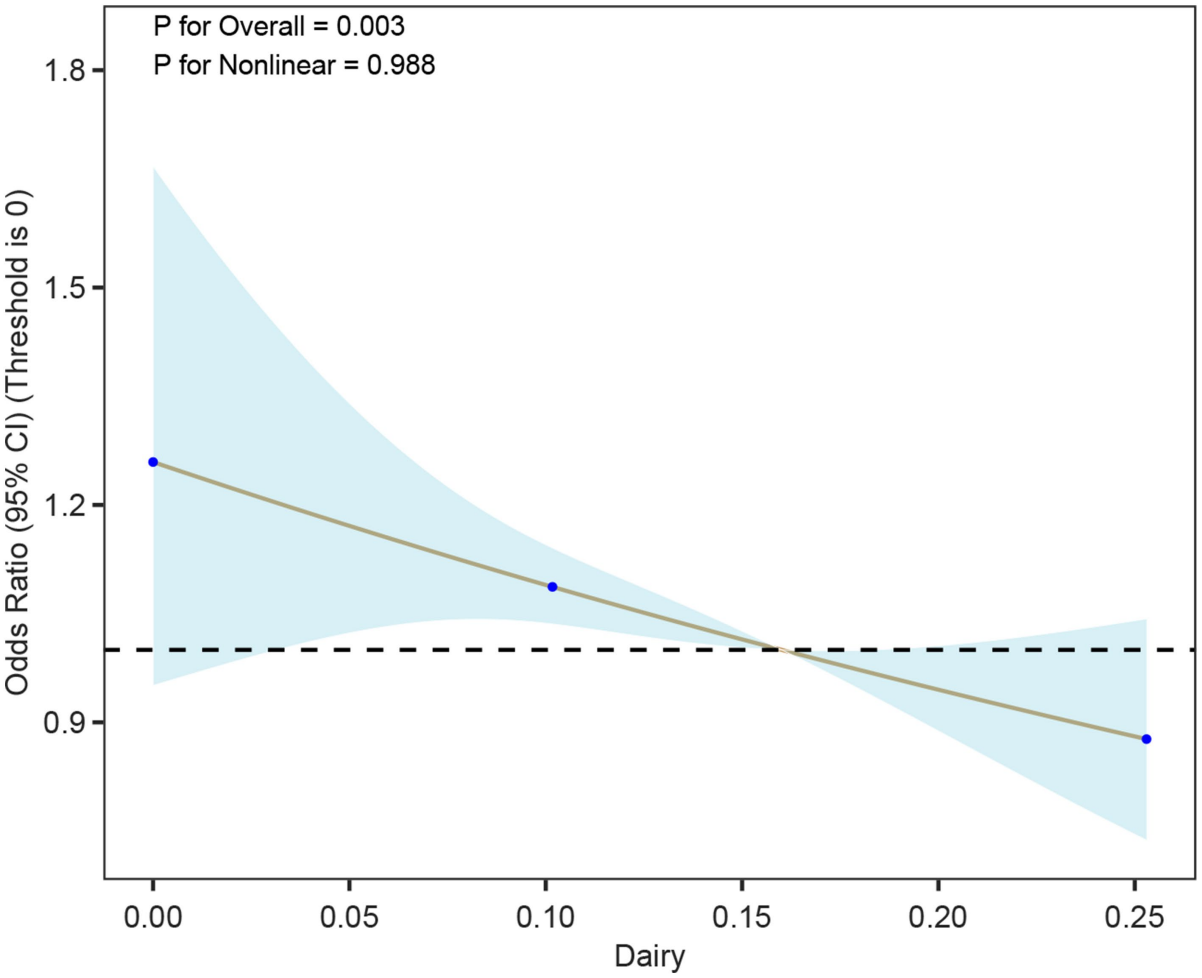
Association between dairy intake and hyperuricemia (HUA) based on restricted cubic spline models. The curve depicts odds ratios (95% CI) across levels of dairy intake, with histograms indicating the distribution of participants.

Using a cohort of a cross-sectional study in southern China for external validation, in Model 1 (unadjusted model), a higher Dairy quantile was significantly negatively correlated with a lower risk of hyperuricemia, especially in the fourth quantile (Quartile 4), the OR was 0.72 (95% CI: 0.59–0.87), and the *p* value was 0.01. In Model 2 and Model 3, after further adjusting for covariates such as age, gender, BMI, smoking, and hypertension, the results were consistent, indicating that Dairy had a continuous protective effect on hyperuricemia, especially in the fourth quartile. More details can be seen in Table 6. The restricted cubic spline plots showed a significant negative correlation between dairy intake and hyperuricemia (Figure 6). As the dairy value increased, the risk of hyperuricemia gradually decreased (*p* < 0.01).

**Table 6:** Association of dairy with HUA.

Variable name	Model 1	*p*-value	Model 2	*p*-value	Model 3	*p*-value
	OR (95%CI)		OR (95%CI)		OR (95%CI)	
Interquartile						
Quartile 1	Reference	-	Reference	-	Reference	-
Quartile 2	0.86 (0.68–1.09)	0.21	0.87 (0.68–1.11)	0.28	0.87 (0.68–1.11)	0.14
Quartile 3	0.80 (0.62–1.04)	0.10	0.81 (0.62–1.06)	0.13	0.81 (0.62–1.07)	<0.01
Quartile 4	0.72 (0.59–0.87)	<0.01	0.73 (0.60–0.88)	<0.01	0.73 (0.60–0.89)	<0.01
P for trend		<0.01		<0.01		<0.01

Model 1, No covariates were adjusted; Model 2, Adjusted for gender and age; Model 3, Adjusted for age, gender, BMI, smoking, and hypertension. HUA, hyperuricemia; OR, Odds Ratio; CI, Confidence Interval.

### 3.6 Mediation analysis

**Figure 7:**
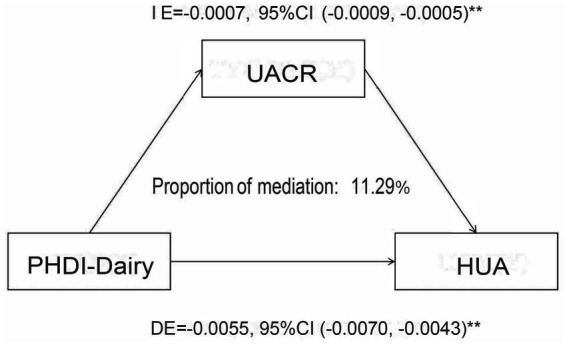
Mediation analysis of the association between PHDI-Dairy and hyperuricemia (HUA) with urinary albumin-to-creatinine ratio (UACR) as a mediator. Indirect effect (IE), direct effect (DE), and proportion mediated were estimated using mediation models with 95% confidence intervals.

In this study, we conducted a mediation analysis to explore the mechanism by which the Planetary Health Diet Index (PHDI-Dairy) influences hyperuricemia (HUA). The results showed that the urinary albumin-to-creatinine ratio (UACR) plays a significant mediating role in the relationship between PHDI-Dairy and HUA. Specifically, the indirect effect (IE) of PHDI-Dairy on HUA was −0.0007, with a 95% confidence interval (CI) of (−0.0009, −0.0005), indicating that PHDI-Dairy exerts its effect on lowering HUA risk through the improvement of UACR. Additionally, the proportion of mediation by UACR was 11.29%, meaning that 11.29% of the effect of PHDI-Dairy on HUA is mediated through the improvement of UACR. More details can be seen in Figure 7.

## 4 Discussion

Based on the nationally representative NHANES data, this study systematically explored the relationship between PHDI-US and the risk of hyperuricemia for the first time. Our analysis showed that a higher PHDI-US score (indicating a healthier plant-based diet pattern) was associated with a significantly reduced risk of HUA. This negative correlation is still robust after a multivariate adjustment model. Although PHDI-US showed a protective effect, its correlation strength was significantly weaker than AHEI and AEI. In order to further optimize the prediction performance, this study applied machine learning methods to improve the PHDI-Dairy index. The association between this index and HUA risk reduction was significantly stronger than that of the original PHDI-US, AHEI, AEI, and MEDI. Sensitivity analysis showed the enhancement effect of PHDI-Dairy, especially its improvement in discrimination and negative predictive value. External validation further confirmed the protective effect of Dairy on hyperuricemia. This finding reveals the importance of weight improvement of the dietary index, and also provides a new idea for dietary intervention of metabolic diseases.

Existing evidence indicates that the PHDI-US is not only associated with a lower risk of HUA but also exhibits protective effects against various chronic diseases. In cardiovascular disease, adherence to PHDI-US is linked to a reduced risk (10), potentially mediated through several mechanisms: dietary fiber moderates glucose absorption into the bloodstream (21); plant-based diets help maintain long-term healthy weight, thereby decreasing the risk of cardiovascular disease and type 2 diabetes (22); and improved gut microbiota composition may influence disease susceptibility (23). In respiratory health, higher PHDI-US adherence is significantly associated with lower asthma prevalence, a correlation partly mediated by BMI (11). For metabolic liver conditions such as Metabolically Dysregulated-Associated Steatotic Liver Disease (MASLD), PHDI’s plant-based orientation, rich in unsaturated fatty acids, may counter the pro-steatotic effects of saturated fats, thereby reducing disease risk (24). In mental health, PHDI-US shows a strong negative correlation with depression, potentially due to limited intake of red meat and saturated fats. Such restriction may reduce reactive oxygen species (ROS) production and oxidative stress, mitigating immune dysregulation and inflammatory responses linked to depression (25, 26). These cross-disease findings suggest that PHDI’s plant-based, animal-limited dietary pattern may exert multi-disease preventive effects through shared metabolic pathways, such as anti-inflammatory and antioxidant mechanisms, offering new insights into managing HUA and metabolic comorbidities.

From the perspective of metabolic pathways, the plant-based diet advocated by PHDI-US can directly reduce the dietary purine load by replacing the red meat diet (27). Intestinal dysbiosis can impair the gut mucosal barrier, triggering low-grade systemic and renal inflammation, thereby elevating serum uric acid levels (28). Phytochemicals exert anti-inflammatory and epigenetic modulatory effects that influence gut flora (29). Sulforaphane (SFN) from cruciferous vegetables inhibits xanthine oxidase and adenosine deaminase activities, thereby reducing uric acid synthesis. It also promotes uric acid excretion by upregulating ABCG2 and downregulating URAT1/GLUT9, while suppressing NLRP3 inflammasome activation via the TLR4/MyD88 pathway (30). Cereal-derived dietary fiber moderates uric acid levels by inhibiting the elevation of serum uric acid induced by dietary RNA, AMP, adenosine, or adenine (31). Furthermore, it enhances glucose-dependent insulinotropic polypeptide (GIP) secretion and improves insulin sensitivity, counteracting hyperuricemia caused by insulin resistance (32). In addition, the regulation of dietary fiber on uric acid may also be affected by inflammatory response, and its detailed mechanism still needs to be further explored (33, 34). Notably, although legumes—particularly soy products—contain moderate to high purine levels and are often restricted in gout diets in Asian countries, a large-scale Singapore Chinese Health Study (n = 63,257) found that both soy and non-soy legume consumption were associated with a reduced risk of gout. Although soy protein may mildly increase uric acid, typical Asian consumption levels are not clinically significant. Multiple studies support that plant-based diets do not elevate hyperuricemia or gout risk, even with high-purine plant foods. Accordingly, the British Rheumatology Association guidelines encourage soy and plant protein intake while advising avoidance of high-purine animal foods (35).

Recent evidence indicates that the controversy surrounding beans and purines in the context of plant-based diets and uric acid metabolism may be overstated. While beans and soy products are relatively rich in purines, several large-scale prospective cohort studies and meta-analyses have consistently shown that plant-derived purines exert a weaker impact on serum uric acid levels and gout risk compared with animal-derived purines such as those from meat and seafood (36). For example, a case-crossover study demonstrated that high intake of animal purines markedly increased the risk of recurrent gout attacks, whereas the effect of plant purines was minimal. In addition, a recent systematic review concluded that habitual consumption of soy products was not associated with an increased incidence of hyperuricemia and might even confer a protective effect (37). Randomized controlled feeding trials, such as those examining the DASH diet, further support that dietary patterns emphasizing plant-based foods modestly lower serum uric acid (38). Current clinical guidelines also reflect this updated understanding: the 2020 American College of Rheumatology guideline and the 2022 NICE guideline both recommend focusing on limiting animal-based purine-rich foods, alcohol, and fructose-containing beverages, while not specifically restricting beans or soy products (39). Similarly, the Chinese dietary guideline for hyperuricemia emphasizes balanced nutrition and preparation methods (e.g., boiling and discarding broth to reduce purine content) rather than avoidance of legumes (40). Collectively, these findings support the inclusion of beans and soy products within plant-based dietary patterns aimed at reducing hyperuricemia risk.

Notably, this study used machine learning to optimize the PHDI-US into PHDI-Dairy. The effectiveness of this improved strategy was shown in the logistic regression model, and external validation further confirmed the significant protective role of dairy against HUA, underscoring the scientific value of targeted dietary index refinement. The original PHDI-US showed weaker protective effects than other indices, likely due to its stronger emphasis on “environmental sustainability.” For example, limiting red meat intake to no more than 2.4% of total calories, but the weight distribution of key nutritional components of uric acid metabolism (such as dairy products) is insufficient. Dairy is a major dietary source of calcium and contains proteins (mainly casein and whey), fat, and lactose. The enhanced performance of PHDI-Dairy may be attributed to several mechanisms: facilitating calcium-dependent urinary uric acid excretion (41); Improving hyperuricemia through whey-derived peptide Pro-Glu-Trp by enhancing intestinal uric acid excretion, modulating gut microbiota, and protecting intestinal barrier in rats (42); and suppressing HUA-related inflammation. Specifically, dairy compounds like GMP and G600 milk fat extract can inhibit IL-1β-mediated inflammation triggered by monosodium urate crystals (43). Our results are further supported by previous population-based studies. A randomized crossover trial reported that consumption of various types of milk reduced serum urate by approximately 10% (44). Higher intake of whole-fat dairy, milk, low-fat dairy, yogurt, and cheese is also associated with decreased hyperuricemia risk (45). These findings suggest that including more dairy and key nutrients in dietary advice can better prevent high uric acid. It also helps create chronic disease strategies that combine nutrition, metabolism, and environmental goals.

From the perspective of clinical significance, the PHDI-Dairy proposed in this study provides a new intervention direction for the prevention and treatment of HUA. In the face of patients with hyperuricemia, doctors may develop personalized dietary plans based on the dietary patterns recommended by PHDI-US, and assist in disease treatment by adjusting the dietary structure. From the perspective of public health, PHDI-US is of great value in environmental protection. PHDI-US advocates increasing plant food intake and reducing the consumption of animal products (especially red meat), which can reduce agricultural-related environmental damage from the source. Taking animal husbandry as an example, animal-free US agricultural modeling systems can reduce US agricultural greenhouse gases (28%) (46). The promotion of the PHDI-US diet can reduce the demand for meat, thereby reducing the scale of animal husbandry, reducing greenhouse gas emissions, alleviating water resources and land pressure, and protecting biodiversity. Other evidence also suggests that higher PHDI-US is associated with lower greenhouse gas emissions (47). This means that integrating the PHDI-US concept into public health policy formulation may reduce the risk of HUA while promoting environmental protection and achieving a win-win situation between human health and the earth’s ecology.

This study has several limitations. The cross-sectional design is difficult to establish the causal timing relationship between dietary patterns and HUA, which needs to be further verified by follow-up cohort studies. PHDI-Dairy’s dairy product weight optimization is based on the dietary characteristics of the American population. Whether it is universal in the Asian population dominated by soybean consumption still needs to be verified by cross-regional research. In addition, the research data relies on 24-h recall, which does not reflect long-term dietary patterns and is susceptible to recall bias. A key limitation of our study is the reliance on two 24-h dietary recalls to estimate usual intake. Although 24-h recalls are widely used in nutritional epidemiology, they may not fully capture long-term dietary habits and are subject to day-to-day variability. Moreover, recall bias is inevitable, as participants may underreport or misreport certain food groups, such as dairy products or plant-based items, which could influence the accuracy of PHDI and dairy intake assessments. These measurement errors may attenuate the observed associations and should be acknowledged as a potential source of uncertainty when interpreting the findings.

Another limitation concerns the generalizability of the optimized PHDI-Dairy. The index was originally derived from U.S. dietary data and subsequently applied to a Chinese cohort, where dietary patterns differ substantially, particularly in the relative contribution of dairy versus soy products. Such cultural differences in staple food choices and protein sources may influence both the validity and applicability of the optimized index across populations. Therefore, while our findings support the utility of PHDI-Dairy in the Chinese context, further validation and potential adaptations are warranted before applying the index in other cultural settings. Future work should consider region-specific dietary structures to ensure broader applicability and accuracy. It should be noted that although the associations between PHDI-US, PHDI-Dairy and health outcomes were statistically significant, their predictive performance in terms of discrimination was relatively weak, as reflected by AUC values close to 0.5. It is important to note that the primary goal of this study was not prediction, but rather to optimize the index. The higher AUC after adjustment compared to before adjustment demonstrates that the optimization was successful. Future research should therefore aim to integrate PHDI-US with other biomarkers or lifestyle indicators to improve predictive utility and to explore its application in diverse clinical and public health contexts. Another limitation relates to the external “southern China” cohort used for validation. Although this cohort provides valuable data for testing the applicability of the index in a different setting, its recruitment procedures and representativeness may limit generalizability. Participants were drawn from a specific regional population, which may not fully reflect the broader demographic and dietary diversity of southern China. In addition, potential selection bias and unmeasured confounding cannot be ruled out, which should be taken into account when interpreting the external validation results.

This study highlights the health benefits of optimizing dairy intake, such as improving bone health and providing essential nutrients like calcium and protein. However, the environmental impact of dairy production, particularly in terms of greenhouse gas emissions and water usage, remains a key concern. Balancing these benefits with sustainability is crucial. One approach is to promote lower-fat or fermented dairy products, which can reduce health risks while still delivering nutritional value. Additionally, incorporating plant-based alternatives, like almond or oat milk, can help reduce environmental impact while providing similar nutritional benefits. Ultimately, optimizing dairy intake in a way that promotes both health and sustainability is central to achieving the goals of the Planetary Health Diet Index.

The mediation analysis in this study revealed that the Planetary Health Diet Index (PHDI-Dairy) influences hyperuricemia (HUA) partly through the improvement of kidney function, as indicated by the urinary albumin-to-creatinine ratio (UACR). Specifically, PHDI-Dairy reduces the risk of HUA by improving UACR levels, accounting for 11.29% of the total effect. This finding underscores the potential role of kidney function in the protective effect of PHDI-Dairy on HUA. Although we did not initially hypothesize that this effect would primarily be mediated through kidney function, this finding offers new insights, suggesting that protecting kidney function may be an important pathway in reducing HUA risk while optimizing diet. Future studies could further explore other potential mediating mechanisms and assess their applicability across different populations.

Based on the above findings, future studies can further carry out prospective intervention trials of PHDI-Dairy to clarify the dose-effect relationship between dairy intake and uric acid metabolism. It is also possible to develop improved versions of regionalized PHDI (such as the Asian version to increase the weight of soy products) to promote the precise and personalized development of dietary intervention programs. These explorations will not only deepen the scientific understanding of the relationship between diet and uric acid metabolism, but also provide a more solid evidence base for the collaborative intervention strategy of `healthy diet-disease prevention-environmental protection`.

## 5 Conclusion

In conclusion, this study suggests that while PHDI-US shows a weaker protective effect against hyperuricemia (HUA) compared with other dietary indices in high-adherence populations, the optimized PHDI-Dairy demonstrates superior performance, providing preliminary evidence to improve the applicability of the EAT-Lancet dietary pattern in individuals with HUA.

## Data Availability

Publicly available datasets were analyzed in this study. This data can be found at: https://www.cdc.gov/nchs/nhanes/.

## Footnotes

^1^ https://www.cdc.gov/nchs/nhanes/index.htm

^2^ https://github.com/jamesjiadazhan/dietaryindex
